# Quality Management of Inert Material During Fluidized Bed Combustion of Biomass

**DOI:** 10.3390/ma19020288

**Published:** 2026-01-10

**Authors:** Marta Wesolowska, Krystian Wisniewski, Jaroslaw Krzywanski, Wojciech Nowak, Agnieszka Kijo-Kleczkowska

**Affiliations:** 1 Department of Regional Studies, University of Lodz Branch in Tomaszow Mazowiecki, Konstytucji 3 Maja 65/67, 97-200 Tomaszow Mazowiecki, Poland; martawesolowska7@gmail.com (M.W.); krystian.wisniewski@filia.uni.lodz.pl (K.W.); 2 Faculty of Science and Technology, Jan Dlugosz University in Czestochowa, Armii Krajowej 13/15, 42-200 Czestochowa, Poland; 3 Faculty of Energy and Fuels, AGH University, A. Mickiewicza 30, 30-059 Krakow, Poland; wnowak@agh.edu.pl; 4 Faculty of Mechanical Engineering, Czestochowa University of Technology, Dabrowskiego 69, 42-201 Czestochowa, Poland; a.kijo-kleczkowska@pcz.pl

**Keywords:** circulating fluidized bed, biomass combustion, bed material, particle size distribution, agglomeration, CFD modeling, bed material regeneration

## Abstract

Fluidized bed combustion of biomass requires maintaining stable properties of the inert bed material, which plays a key role in heat transfer, temperature stabilization and uniform fuel distribution in circulating fluidized bed (CFB) boilers. During long-term operation, quartz sand, i.e., the most commonly used inert material, undergoes physical and chemical degradation processes such as attrition, sintering and coating with alkali-rich ash, leading to changes in particle size distribution (PSD), deterioration of fluidization quality, temperature non-uniformities and an increased risk of bed agglomeration. This study analyzes quality management strategies for inert bed materials in biomass-fired CFB systems, with particular emphasis on the influence of PSD on boiler hydrodynamics and thermal behavior. Based on industrial operating data, sieve analyses and CFD simulations performed under representative operating conditions, a recommended mean particle diameter range of approximately 150–200 μm is identified as critical for maintaining stable circulation and uniform temperature fields. Numerical results demonstrate that deviations toward coarser bed materials significantly reduce solids circulation, promote segregation in the lower furnace region and lead to local temperature increases, thereby increasing agglomeration risk. The study further discusses practical approaches to bed material monitoring, regeneration and make-up management in relation to biomass type and ash characteristics. The results confirm that systematic control of inert bed material quality is an essential prerequisite for reliable, efficient and low-emission operation of biomass-fired CFB boilers.

## 1. Introduction

Biomass combustion in circulating fluidized bed (CFB) systems has gained increasing attention as a viable pathway for sustainable energy production due to its high combustion efficiency, broad fuel adaptability and favorable emission performance, particularly when compared to conventional combustion technologies [1]. Modern CFB boilers can efficiently process diverse biomass fuels, ranging from woody residues to agricultural wastes, while maintaining stable temperature profiles and achieving effective pollutant reduction through enhanced gas–solid mixing and recirculation dynamics [2]. Recent research also highlights that CFB technology promotes uniform heat transfer and stable hydrodynamics under various operating conditions, supporting reliable operation in industrial-scale energy systems [3]. Despite these advantages, the operational performance and long-term reliability of biomass-fired CFB boilers remain strongly dependent on the properties and management of the inert bed material, which directly affect fluidization behavior, heat transfer and the risk of agglomeration during combustion [1,4].

The management of the supplementary material (sand) used together with biomass in circulating fluidized bed boilers (CFB) is essential to ensure the stable and efficient operation of the power unit. Proper operation of the combustion system requires a continuous supply of sand with a well-defined particle size distribution. When sand is employed as the inert bed material, its granulometric characteristics must meet the specifications recommended by the boiler manufacturer [5,6].

The grains are typically of a rounded shape, which ensures smooth fluidization and minimizes mechanical wear of internal components. However, the presence of angular particles can intensify erosive effects on the heat exchange surfaces and refractory linings within the combustion chamber. Such erosion may lead to accelerated degradation of the boiler’s internal elements, changes in fluidization behaviour, and ultimately, a reduction in unit efficiency. Therefore, systematic quality control of the bed material covering both its physical properties (grain shape, size and hardness) and chemical purity is a key aspect of operational management in fluidized bed biomass combustion systems [7,8].

The determination of the particle size distribution involves separating the sand material into fractions containing grains of different sizes by means of sieving through a series of meshes with progressively smaller openings. As the sample is passed through the sieve stack, grains of corresponding diameters are retained on successive sieves, allowing a physical separation of the material by size. Each fraction is then weighed and the percentage of material retained on each sieve is calculated relative to the total sample mass.

The resulting data are used to construct a particle size distribution curve (PSD), which provides a graphical representation of the granulometric composition of the sand. This curve is plotted on a semi-logarithmic grid, with grain diameter on the logarithmic axis and the cumulative percentage (or percentage retained) on the linear axis. The smooth, continuous curve obtained from this analysis characterizes the gradation of the sand and allows for the assessment of its suitability as an inert bed material.

A well-graded sand featuring an optimal proportion of fine and coarse fractions ensures stable fluidization, uniform heat transfer, and efficient combustion conditions in the fluidized bed. Conversely, deviations in the particle size distribution, such as an excess of fine particles or the presence of oversized grains, may lead to channeling, slugging, or uneven fluidization, all of which negatively affect boiler performance and increase operational wear [9].

In fluidized bed boilers firing biomass, the inert bed material (most often quartz sand) ensures proper heat transfer, temperature uniformity, and fuel mixing. Its quality deteriorates over time due to attrition, sintering, and especially coating with alkali-rich ash layers originating from the fuel. These mechanisms alter the bed’s thermal activity and hydrodynamics, leading to defluidization or agglomeration [10]. This phenomenon has been extensively studied and classified into two main mechanisms: coating-induced and melt-induced agglomeration, both depending on bed temperature, alkali content in ash, and sand composition [9,10]. The material’s degradation directly affects boiler stability, efficiency, and emission control. Periodic PSD monitoring helps identify “particle drift” caused by attrition, sintering, or agglomeration during operation. Shifts toward coarser fractions have been correlated with overheating episodes and coating-induced fusion [11].

In biomass-fired systems, bed material degradation is accelerated by chemical interaction between silica and alkali elements (mainly K and Na) released during combustion. These species react with quartz to form low-melting potassium silicates (K_2_O·nSiO_2_), which subsequently promote the adhesion and coalescence of sand particles [12]. With time, these silicate coatings grow thicker, decreasing the particle’s specific surface area and altering the bed’s fluidization behaviour.

At higher temperatures, melt-induced agglomeration may occur due to the formation of eutectic K–Ca–Si phases. The addition of calcium-containing minerals or the natural presence of CaO can partially stabilize these coatings by forming compounds with higher melting points, reducing stickiness and delaying defluidization [13]. Detailed thermodynamic studies and SEM/EDS analyses have confirmed that the morphology and composition of coatings evolve with operating temperature and the chemical makeup of the fuel ash. Numerous studies have explored substitutes for quartz sand to mitigate alkali–silica reactions. Olivine, ilmenite, feldspar, and blast furnace slag show better resistance to coating formation and improved catalytic activity for tar cracking or char oxidation. Recent comparative experiments [14] demonstrated that K-feldspar and olivine accumulate less alkali coating than quartz under similar conditions in bubbling fluidized bed (BFB) tests. Their higher melting points and lower chemical reactivity make them attractive for long-term operation in biomass boilers and gasifiers.

Advancements in diagnostic technologies have enabled real-time monitoring of bed material quality. Common approaches include SEM/EDS analysis of coating morphology, on-line and off-line PSD measurements, and analysis of pressure fluctuation spectra to detect early signs of agglomeration. Acoustic and impedance-based sensors have also been proposed to assess coating growth and bed heterogeneity [15]. Recent developments in computational fluid dynamics (CFD) and machine learning provide new tools for predictive maintenance. Euler–Euler multiphase models can simulate bubble dynamics, heat transfer, and agglomeration thresholds based on real operating data, offering early warning of defluidization events [15,16].

While the primary focus of this study is the energetic utilization of biomass in Circulating Fluidized Bed (CFB) boilers, it is essential to situate this process within the broader framework of the circular economy and sustainable resource management. Biomass is increasingly recognized as a versatile precursor for high-value applications beyond direct combustion. For instance, recent advancements in materials science demonstrate the potential of modified biomass (such as cellulose, lignin, and starch) in reinforcing polylactic acid (PLA) composites to enhance mechanical and thermal properties [17]. Similarly, waste biomass can be valorized through advanced synthesis to produce carbon quantum dots for environmental sensing applications [18] or converted via pyrolysis and composting into biochar and organic fertilizers for soil amelioration [19]. Despite these alternative valorization pathways, the energetic conversion of biomass remains a cornerstone of net-zero emission strategies, particularly for stabilizing power grids and managing non-recyclable organic fractions. Consequently, ensuring the efficiency of this combustion process through rigorous quality management of the inert bed material is critical to maintaining operational stability and preventing alkali-induced agglomeration.

It should be emphasized that the introduction of fluidized bed combustion technology has primarily enabled the reduction in gas emissions and the combustion of lower-quality fuels, including waste fuels generated during coal enrichment [20,21,22]. Fluidized bed boilers achieve these results primarily through low temperatures of combustion (800–950 °C), multi-stage and cyclic combustion, combustion chamber–reversal system–combustion chamber (in the case of the circulating fluidized bed boilers CFB), leading to a significant reduction in nitrogen oxides, the addition of sorbents (dolomite and limestone) binding sulfur oxides by up to 90%, the characteristic flow of bulk material, which allows for a high degree of substrate mixing, and the high intensity of heat and mass transfer processes [20,21,22].

In biomass-fired CFB boilers, intrinsic fuel properties such as particle size distribution, moisture content, and bulk density significantly influence combustion behavior, ignition characteristics, devolatilization and the combustion efficiency. Studies have shown that fuel densification and particle size variations affect ignition, burnout, and emission characteristics while moisture and bulk density modify heat transfer conditions and reaction rates during combustion [23,24]. Although these parameters strongly impact combustion performance and pollutant formation, the present study focuses on the management of the inert bed material, which governs fluidization dynamics, heat transfer, and long-term operational stability of the fluidized bed under varying fuel qualities.

In this article, the focus is placed on the particle size distribution (PSD) of the inert bed material and its influence on the stable operation of a fluidized bed boiler. The PSD determines the hydrodynamic behaviour of the bed, affecting fluidization quality, mixing efficiency, heat transfer, and the overall combustion process. Deviations from the recommended granulometric range can lead to uneven temperature profiles, increased erosion of heat transfer surfaces, reduced combustion efficiency and even partial defluidization [25]. Maintaining a PSD consistent with the boiler manufacturer’s specifications is therefore essential for long-term reliability and efficiency of CFB operation. An optimal grain size distribution ensures uniform air flow through the bed, stable bubble formation, and homogeneous fuel combustion. Conversely, the accumulation of fine or oversized fractions during operation changes the fluidization regime and increases operational risks. For these reasons, continuous monitoring and periodic adjustment of the PSD are integral elements of effective quality management of the bed material in fluidized combustion systems.

In the present study, the investigated system consists of a circulating fluidized bed boiler fired with solid biomass fuels representative of industrial practice. The inert bed material considered in both experimental observations and numerical simulations is quartz sand, treated as a single solid phase with a defined particle size distribution. The fuel is introduced as solid biomass particles, while the gas phase consists of air and combustion products. No chemical speciation of gaseous products is explicitly resolved; instead, the focus is placed on the physical behavior of solid phases, particularly the particle size distribution of the inert bed material and its impact on fluidization stability.

## 2. Recommended PSD of the Inert Bed Material

When ordering inert bed material (sand) for use in a fluidized bed boiler, it is essential to adhere strictly to the PSD recommended by the CFB boiler manufacturer.

As an example, the sand should meet the following PSD requirements:

- 100% of particles below 600 μm;
- 75% below 250 μm;
- 50% below 180 μm;
- 25% below 130 μm.

**Figure 1:**
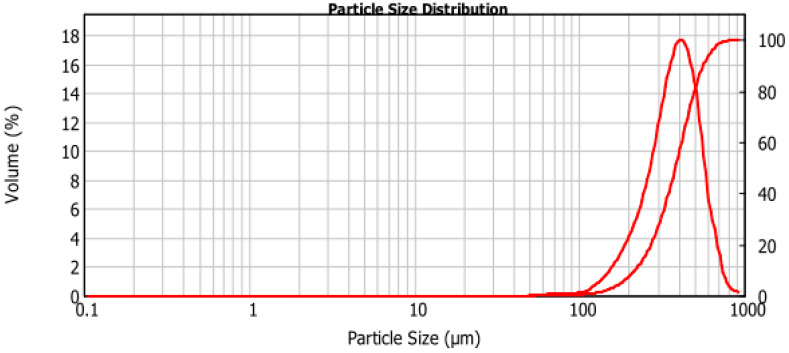
An example of typical particle size distributions of sand delivered for CFB unit.

Such specifications ensure an optimal balance between fine and coarse fractions, providing uniform fluidization, stable temperature distribution across the bed and effective combustion of biomass particles. Sand that is too coarse can result in uneven air distribution and higher erosion rates, whereas excessive fines may lead to entrainment losses, unstable fluidization and the risk of bed agglomeration. Figure 1 presents an example of typical particle size distributions of sand delivered for a CFB unit. These data illustrate the importance of maintaining a consistent PSD within the specified limits throughout operation. Continuous monitoring of the supplied sand through sieve analysis and routine inspection helps ensure that the material complies with technical requirements and sustains optimal performance of the circulating fluidized bed system.

**Figure 2:**
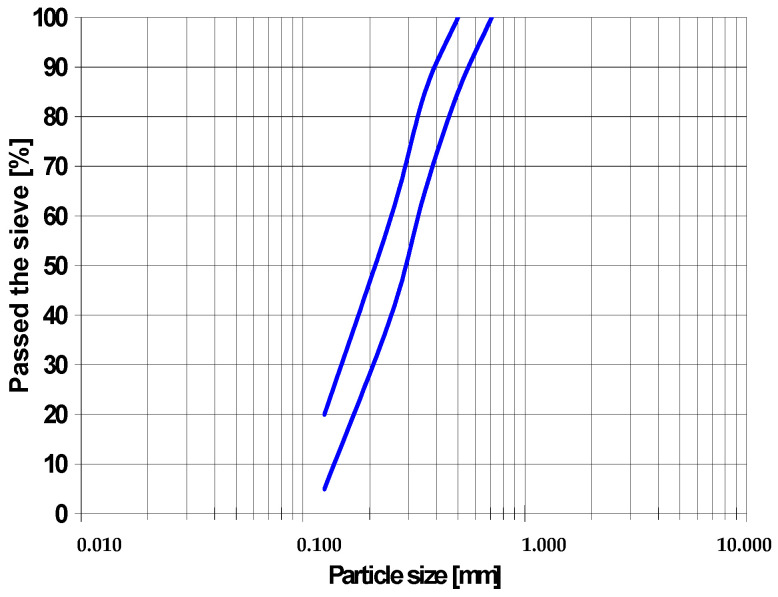
An example of PSD of sand.

The PSD of the inert bed material can also be presented graphically, as shown in Figure 2, where the cumulative distribution curve illustrates the percentage of particles passing through successive sieve sizes. This graphical representation makes it possible to visualize the range and uniformity of grain sizes in a more intuitive way, highlighting both fine and coarse fractions. Such a presentation allows quick verification that the supplied material meets the CFB boiler manufacturer’s requirements and facilitates comparison between different batches of sand or between fresh and used bed material during operation. These particle size distributions are specified because they are essential for the proper operation of the CFB boiler.

The analysis presented in this study is based on deterministic evaluation of operational and granulometric data obtained from industrial CFB boiler operation. No formal statistical hypothesis testing was applied, as the objective was to identify physically meaningful trends in particle size distribution and fluidization behavior rather than to perform probabilistic inference. Measurement uncertainty inherent to sieve-based PSD determination is estimated to be within ±2–5%, which does not affect the qualitative interpretation of the observed trends. The reported results therefore reflect engineering-relevant changes in bed material behavior.

If the input materials to the CFB boiler (sand, fuel, and limestone) are out of specification and, as a result, the particle size distribution is also far from the design values, then serious problems can be expected with the operation of the CFB boiler, including higher temperatures, higher emissions, sintering, etc. The temperature control in the combustion chamber will be satisfactory only when the PSD of the bed is consistent with the assumed one. When sand particles are too coarse, higher bed temperatures result due to lower heat transfer in the combustion chamber.

## 3. Influence of Inert Material Quality on CFB Boiler Performance

Maintaining stable operating conditions in a circulating fluidized bed (CFB) boiler requires effective control of temperature fluctuations within the furnace chamber and the avoidance of vertical temperature non-uniformities along its height. These parameters directly affect combustion efficiency, emission levels, and the durability of boiler components. One of the most important factors influencing the thermal and hydrodynamic stability of the system is the quality of the inert bed material, particularly its particle size distribution (PSD) and physical characteristics. Ensuring that the supply of sand meets the granulometric specifications recommended by the CFB boiler manufacturer is essential for sustaining proper fluidization, uniform mixing of fuel particles, and consistent heat transfer within the bed. Deviations from the optimal PSD can result in uneven temperature fields, unstable circulation of solids and impaired combustion performance. Fine particles tend to elutriate from the bed, causing loss of mass and instability, whereas excessively coarse grains can reduce mixing intensity and contribute to the formation of hot spots or localized sintering.

It can therefore be stated, with full technical confidence, that the appropriate selection and control of the inert material, including its size distribution, purity and mechanical integrity, plays a decisive role in the efficient and reliable operation of biomass-fired CFB boilers. Continuous monitoring of sand quality and systematic replacement of degraded material are necessary preventive measures to maintain optimal combustion efficiency and to minimize the risk of agglomeration, erosion and unplanned shutdowns.

Even if the supplied sand initially meets the required particle size range, its composition and granulometry inevitably change during boiler operation. Over time, the original sand particles are gradually replaced by ash-derived particles produced from the fuel combustion. This process is particularly pronounced when burning coal with a high ash content, as the continuous influx of mineral residue partially compensates for sand losses. However, when biomass with a low ash content is used as fuel, this natural replenishment mechanism is negligible. Under such conditions, continuous sand dosing or frequent replenishment becomes necessary to maintain the required bed inventory and granulometric composition.

**Figure 3:**
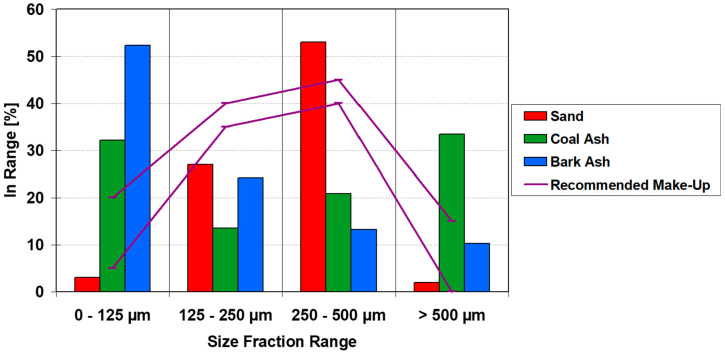
PSD of materials delivered to the CFB boiler.

As an example, this relationship is illustrated in Figure 3, where the solid line represents the recommended the range of PSD for the inert material as specified by the CFB boiler supplier. The term “make-up material” (or simply “make-up sand”) refers to the inert bed material in this case, sand that is periodically added to the fluidized bed to compensate for losses caused by attrition or elutriation during boiler operation. For stable operation, the mean particle diameter of the bed material should remain within the range of 150–200 μm. As shown, neither the ash produced from coal nor from biomass combustion meets these requirements, and even the delivered sand often falls outside the optimal range. This discrepancy clearly indicates improper material selection and inadequate quality management of sand supply.

Selecting and maintaining the correct sand particle size is therefore crucial for the trouble-free operation of a biomass-fired fluidized bed boiler. Failure to preserve the appropriate granulometric distribution can lead to operational instability [26]. Excessively coarse particles tend to accumulate in the furnace chamber, requiring removal through the bottom ash extraction system, while fine particles may be carried out with the flue gas stream, reducing the bed mass and deteriorating heat transfer [27,28]. Both phenomena disturb thermal balance and may result in undesirable temperature increases in the combustion zone, potentially leading to bed agglomeration or localized overheating.

Bed material regeneration in biomass-fired CFB boilers can be classified into mechanical, chemical, and preventive material substitution approaches. From an industrial perspective, mechanical screening remains the most commonly applied regeneration method [9]. By removing oversized agglomerates and excessive fines, screening allows partial restoration of the particle size distribution (PSD) toward the manufacturer-recommended range. This approach is characterized by low capital cost, operational simplicity, and compatibility with continuous boiler operation; however, it does not remove chemically bonded alkali-rich coatings formed on quartz sand.

Chemical regeneration methods, such as acid washing or alkali leaching, are effective in reducing potassium and sodium content and in restoring the surface properties of silica-based bed materials. Laboratory-scale studies demonstrate that these methods can significantly delay agglomeration onset by removing low-melting alkali–silicate phases. Nevertheless, their application at industrial scale is limited due to high operational costs, chemical consumption, wastewater treatment requirements, and the need for off-line processing. Consequently, chemical regeneration is not commonly implemented in utility-scale CFB boilers and is primarily considered for experimental investigations or highly specific operational cases [9,12].

An alternative strategy involves the partial or full replacement of quartz sand with alkali-resistant bed materials such as olivine or potassium feldspar. These materials exhibit lower chemical reactivity with alkali species and higher resistance to coating-induced agglomeration, thereby extending bed material lifetime [13,14]. However, their higher procurement cost and limited availability mean that their application is typically justified only in units firing high-alkali fuels or experiencing recurrent agglomeration problems. In practice, bed material management in biomass-fired CFB systems relies on a combination of mechanical screening, controlled make-up dosing, and fuel quality monitoring rather than on chemical regeneration alone.

**Figure 4:**
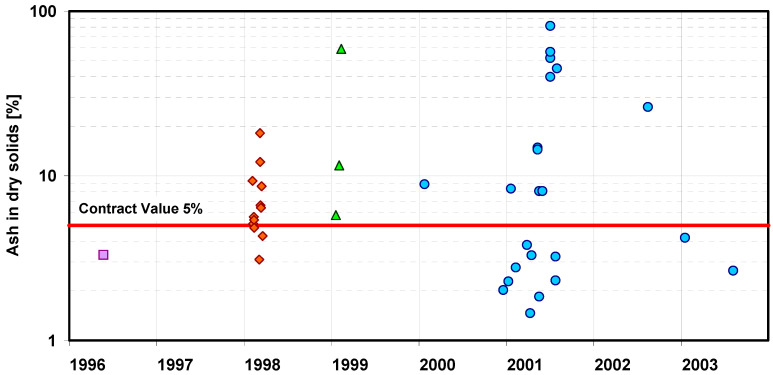
Example of biomass deliveries contaminated with mineral impurities (markers on the graph indicate different fuel deliveries).

Besides the particle size distribution of the inert bed material, the quality of the biomass fuel itself is another critical factor influencing the stability of CFB boiler operation. When managing biomass deliveries, it is equally important to pay close attention to their quality, since the supplied material may contain sand, stones and other mineral impurities, as illustrated in Figure 4. The contracted ash content of the biomass fuel was specified at 5%, yet in practice, the delivered material often contained contamination levels significantly exceeding this contractual limit. Such contamination leads to serious operational disturbances, affecting not only the CFB boiler performance but also the biomass feeding system. The presence of abrasive particles accelerates erosive wear of mechanical components such as screw conveyors, feeders and pneumatic transport lines, resulting in frequent maintenance interventions and premature replacement of worn parts. In the long term, poor control of biomass quality increases downtime, raises maintenance costs and reduces the overall efficiency and reliability of the CFB combustion system.

**Figure 5:**
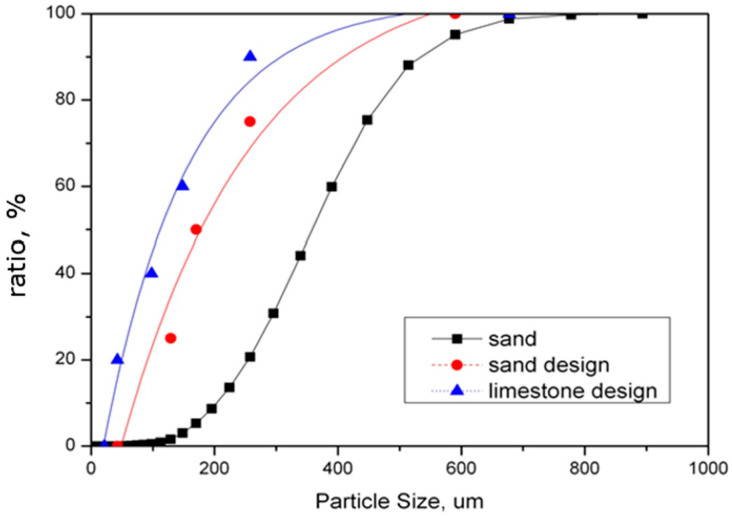
PSD of sand and sorbent—design versus actual.

The particle size distribution of the fluidized bed material is strongly influenced by the fragmentation and attrition behaviour of both ash and sorbent particles. During combustion, these materials undergo mechanical and thermal degradation, gradually modifying the granulometric composition of the bed. In the case of biomass with a low ash content, the bed material is composed almost entirely of sand supplied as make-up material together with the fuel since the contribution of ash particles to the bed mass is minimal. Figure 5 presents the recommended PSDs for both the sand and the sorbent (limestone), as specified by the boiler manufacturer. The figure also includes the actual PSD of the sand sampled from the operating unit, which deviates considerably from the prescribed range. This discrepancy indicates that operational issues observed during biomass combustion may result from an improper sand size distribution, particularly when the sand is too coarse. Coarse particles deteriorate fluidization quality, reduce the efficiency of heat transfer and increase the risk of temperature gradients or local sintering within the furnace chamber.

Moreover, the granulometry of the bed can also be affected by the ash produced during biomass combustion [29]. Monitoring the physicochemical properties of biomass is critical, as commercial fuels often deviate from standardized specifications. Research by Kamperidou [29] on wood pellets demonstrated that actual ash content can significantly exceed certified limits, often due to the presence of impurities such as bark or mineral fines. These unintended mineral loads introduce additional alkali and silica into the furnace, exacerbating the bed agglomeration risks discussed herein and validating the need for continuous bed quality management regardless of the nominal fuel type.

**Figure 6:**
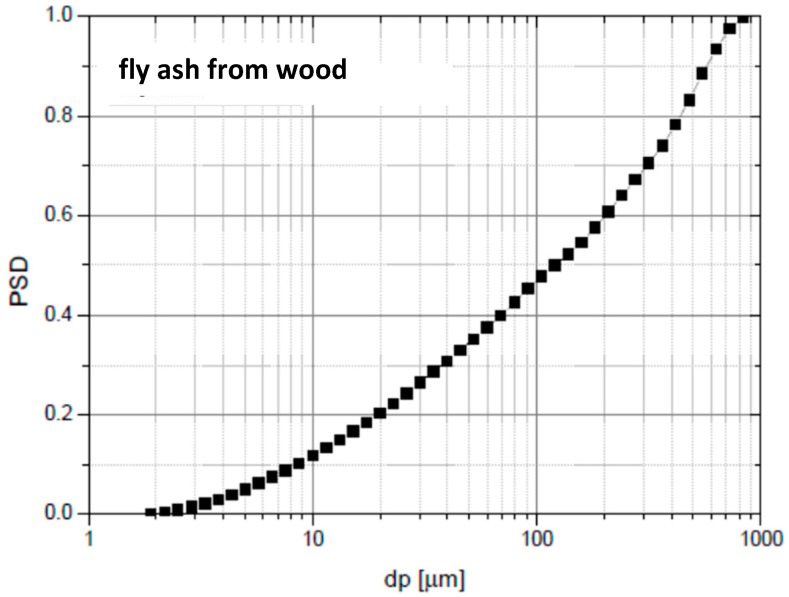
PSD of fly ash produced during the combustion of wood biomass.

Figure 6 illustrates the particle size distribution of ash derived from wood combustion, which typically consists of fine particles. The results correspond to industrial wood chips originating from mixed softwood biomass (mainly pine and spruce), representative of typical fuels used in commercial CFB biomass boilers.

When mixed with the sand bed, these fine ash particles can shift the overall PSD toward smaller sizes, thereby altering the hydrodynamic behaviour of the system and influencing both fluidization stability and separation efficiency in the cyclone loop. Proper monitoring and management of PSD evolution accounting for the combined effects of sand, sorbent attrition, and ash input is therefore essential for maintaining optimal combustion stability and preventing operational disturbances in biomass-fired CFB boilers.

The ash produced from wood combustion is extremely fine, and when exposed to the intense turbulent motion within the fluidized bed furnace, it undergoes further attrition and secondary fragmentation. As a result, the ash particles become even smaller and lighter, easily carried upward by the flue gas stream. Such fine material is unable to participate in the circulation loop of a CFB boiler and therefore does not contribute to the formation of the stable, recirculating bed inventory. Instead, it is entrained from the furnace and separated in the cyclone or collected in downstream filters. Consequently, the inert bed material, that is, the sand supplied together with the biomass fuel, plays a dominant role in determining both the particle size distribution of the fluidized bed and the temperature profile within the combustion chamber. In practice, this means that maintaining proper sand quality and granulometry is essential to ensure stable fluidization, uniform heat transfer and efficient combustion. When the sand is too coarse or insufficiently replenished, the lack of fine recirculating material leads to uneven temperature distribution, localized hot spots and potential operational disturbances within the CFB system.

**Figure 7:**
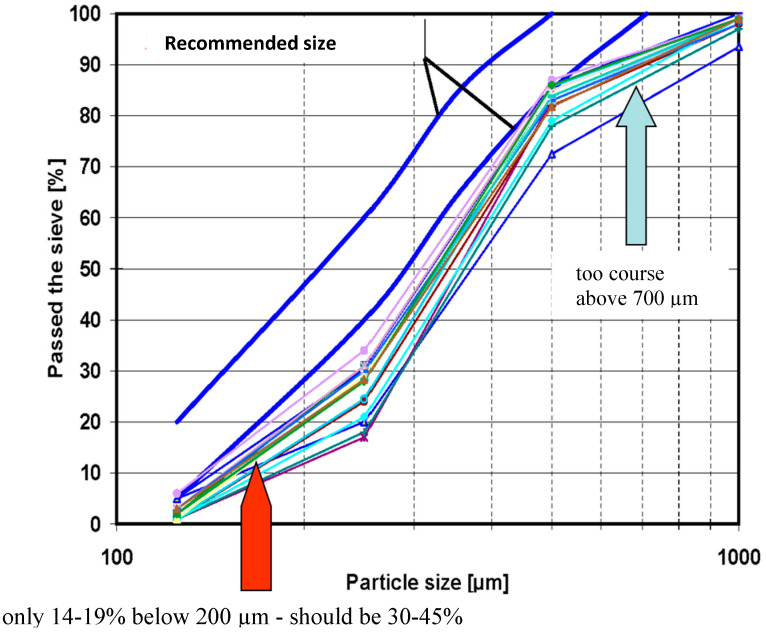
Improper management of make-up sand deliveries to a fluidized bed boiler fired with 100% biomass.

The case presented in Figure 7 refers to the deliveries of sand supplied to a fluidized bed boiler operating with 100% biomass fuel. The recommended particle size distribution range, as specified by the boiler manufacturer, is indicated by the blue line on the graph. As can be seen, the actual PSD curves for the delivered sand consistently fall below the recommended range, indicating that the sand supplied to the boiler contained a higher proportion of fine particles than required. Such material fails to provide the necessary fluidization stability and thermal buffering capacity within the combustion chamber.

It is crucial to differentiate the degradation mechanisms based on the biomass origin. While woody biomass typically possesses a lower alkali content, herbaceous fuels (e.g., straw) are characterized by high concentrations of potassium and sodium, which significantly accelerate the coating formation on quartz grains. From a materials engineering perspective, analogous to surface modifications observed in biomass-based composites or adsorbents [17,18], the ash components chemically modify the surface of the inert material. In the context of CFB combustion, this surface modification leads to the formation of low-melting eutectic silicates (K–Ca–Si systems), creating sticky layers that facilitate bed agglomeration much faster than in wood-fired systems. Understanding these chemical interactions is vital for predicting the lifespan of the bed material.

Ideally, the assessment of these coating kinetics and the precise agglomeration temperature would be supported by microstructural investigations, such as Scanning Electron Microscopy (SEM) coupled with Energy-Dispersive Spectroscopy (EDS). While such advanced characterization techniques are successfully employed in material science to study biomass surface functionalization and elemental distribution [18,19], they were not available for the specific industrial-scale operational cycles analyzed in this study. Consequently, the discussion presented herein relies on macroscopic operational data (PSD drift and temperature profiles) and validated mechanisms from peer-reviewed literature, rather than direct microstructural observation of the specific bed samples.

## 4. Simulations for Managing Make-Up Material (Sand) with Biomass Deliveries in a CFB Boiler

**Figure 8:**
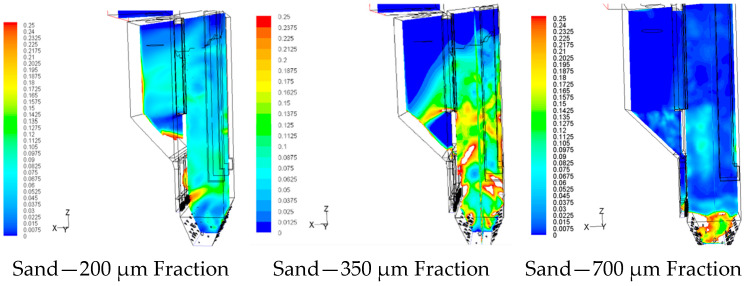
Numerical simulations of the density distribution of bed material in a CFB boiler for various sands supplied to the power plant.

Model simulations of sand supply management and its operational impact were performed in ANSYS CFD (ANSYS, Inc. Ansys Fluent Theory Guide; ANSYS Inc.: Canonsburg, PA, USA, 2013) for a representative circulating fluidized bed (CFB) boiler firing 100% biomass. Operating conditions were selected as typical for this class of units: bed temperature ≈ 850 °C and superficial gas velocity ≈ 5 m/s. The influence of the PSD of the inert bed on the in-furnace structure was analysed. Figure 8 shows the resulting fields. A 3-D Euler–Euler multiphase framework was used with a gas phase (air/flue gas) and a solid phase (sand). Interphase drag followed a standard Wen–Yu/Gidaspow blending; solid–solid and wall restitution coefficients were chosen within literature norms for silica sand at high temperature. Turbulence was captured with a k–ε model on the gas phase; solids turbulence was represented via granular temperature. Radiative heat transfer was included to resolve temperature stratification. PSD cases were applied as mono-modal Sauter-mean diameters representative of delivered blends.

The numerical simulations were intentionally designed as a single-factor parametric study, in which the particle size distribution (PSD) of the inert bed material was varied while other key operating parameters, including biomass feed rate, furnace pressure and superficial gas velocity, were kept constant at values representative of stable industrial CFB operation. This approach allows the isolated assessment of PSD effects on hydrodynamics and temperature fields, avoiding ambiguity associated with multi-parameter coupling. Model predictions were qualitatively validated against long-term operational observations from industrial biomass-fired CFB boilers, including temperature stability, pressure behavior and documented segregation phenomena under coarse bed material conditions. The following cases were considered:

1. Recommended sand: d_50_ ≈ 200 µm (contract specified size of make-up material).
2. Slightly coarser sand: d_50_ ≈ 350 µm (just above the recommended range).
3. Coarse sand: d_50_ ≈ 750 µm (market-driven, clearly outside specification).

The results, illustrated in Figure 8, show a clear dependence of the flow and temperature fields on sand granulometry. For the recommended sand (*d*_50_ = 200 μm), the bed exhibited a stable bubbling-circulating regime with uniform solids concentration and nearly isothermal temperature distribution throughout the combustion chamber. The predicted pressure drop and solids circulation rate were consistent with design values, confirming proper hydrodynamic behaviour.

In contrast, the use of coarser sand (*d*_50_ = 350 μm and 750 μm) caused a noticeable deterioration in fluidization quality. Larger particles reduced solid holdup and intensified bubble coalescence, producing uneven gas–solid mixing and unstable circulation loops. Temperature fields revealed vertical and lateral non-uniformities, with local hot spots up to 40 °C above the nominal bed temperature, particularly in the lower freeboard region. For the coarsest sand (*d*_50_ = 750 μm), the circulation flow significantly decreased and the unit behaviour shifted toward that of a bubbling bed, leading to impaired heat transfer and an increased risk of agglomeration.

The simulation results confirm that compliance with the recommended PSD (mean diameter 150–200 μm) is essential for maintaining stable hydrodynamics, uniform heat transfer, and safe operation of biomass-fired CFB boilers. Deviations toward coarser sands, often driven by market availability, result in measurable deterioration of combustion conditions, higher risk of local sintering and reduced efficiency. The findings clearly indicate that proper make-up sand management including PSD control at delivery and continuous monitoring during operation is a critical factor in ensuring long-term performance and reliability of CFB technology.

The numerical simulations clearly demonstrate that when sand with an average particle size of approximately 700 μm is used as bed material, pronounced segregation occurs within the combustion chamber. In this case, the coarse sand grains accumulate in the lower part of the furnace, while the upper regions become depleted of solids. This uneven distribution leads to distorted gas–solid flow patterns and localized temperature imbalances. Such segregation is highly undesirable, as it results in hot spots and excessive temperature increases in the lower section of the combustion chamber. These conditions can severely disrupt the hydrodynamic stability of the fluidized bed, reduce the efficiency of heat transfer and promote sintering or agglomeration of the bed material. In extreme cases, these effects can escalate into serious operational failures, including partial defluidization or complete CFB boiler shutdown.

Therefore, it must be emphasized that the acceptance of sand with a mean particle size of approximately 700 μm is inconsistent with the manufacturer’s specifications and substantially increases the operational risk associated with unstable fluidization. Maintaining the proper granulometric specification of the inert bed material typically around 200 μm is essential to ensure safe, efficient and reliable operation of the fluidized bed combustion system.

## 5. Conclusions

Based on the obtained results, the following key findings and their direct implications for the operation of CFB boilers, with particular emphasis on the role of inert bed material particle size distribution, can be summarized.

- Numerical simulations and operational observations demonstrate that, in biomass-fired CFB boilers, the particle size distribution (PSD) of the inert bed material plays a decisive role in shaping the furnace temperature profile. In particular, for low-ash biomass fuels, appropriate sand granulometry ensures uniform gas–solid mixing, stable heat transfer, and reduced thermal stress on boiler components.
- CFD simulations indicate that the use of overly coarse sand (mean particle size above 350–400 μm) leads to deterioration of fluidization quality, segregation of bed material near the distributor, and localized overheating. Such conditions promote defluidization and agglomeration, confirming that compliance with the recommended PSD range (d_50_ ≈ 150–200 μm) is essential for safe and stable CFB operation.
- The results confirm that, in biomass-fired CFB boilers, the circulating bed inventory cannot be sustained by ash alone and requires systematic make-up sand addition. Continuous monitoring of PSD and controlled replenishment are therefore necessary to maintain hydrodynamic stability and steady combustion conditions.
- The study shows that variations in sand quality from different suppliers have a direct and measurable impact on combustion efficiency, operational stability, and component lifetime in CFB boilers.
- Maintaining a narrow and well-defined PSD of the inert bed material, supported by continuous monitoring and CFD-based analysis, enables improved control of make-up dosing strategies, mitigation of agglomeration risk, and extension of boiler service life.

## Data Availability

The original contributions presented in this study are included in the article. Further inquiries can be directed to the corresponding author.
